# Landing-Energy-Controlled Surface Conformation of Electrosprayed Foldamer Molecules on Au(111)

**DOI:** 10.1021/acsnano.5c12973

**Published:** 2026-01-18

**Authors:** Shengming Zhang, Dennis Meier, Patrick Lawes, Pengfei Zhao, Jinhua Wang, Victor Maurizot, Andreas Walz, Annette Huettig, Hartmut Schlichting, Anthoula C. Papageorgiou, Joachim Reichert, Ivan Huc, Johannes V. Barth

**Affiliations:** † Physics Department, E20, TUM School of Natural Sciences, 9184 Technical University of Munich, 85748 Garching, Germany; ‡ Department of Chemistry, 158259 Tufts University, Medford, Massachusetts 02155, United States; § Institute of Nanotechnology and Institute of Quantum Materials and Technology (IQMT), 150232 Karlsruhe Institute of Technology, 76131 Karlsruhe, Germany; ∥ Institut de Physique et de Chimie de Matériaux (IPCMS), Université de Strasbourg, UMR 7504, F-67034 Strasbourg, France; ⊥ CNRS, Bordeaux INP, CBMN, UMR5248, Univ. Bordeaux, F-33600 Pessac, France; # pureions GmbH, 82205 Gilching, Germany; ∇ Laboratory of Physical Chemistry, Department of Chemistry, 68993 National and Kapodistrian University of Athens, Athens 157 71, Greece; ○ Department of Pharmacy, Ludwig-Maximilians-University Munich, 81377 Munich, Germany; ◆ Cluster of Excellence e-conversion, 85748 Garching, Germany

**Keywords:** electrospray ionization, scanning tunneling microscopy, foldamer molecules, landing energy, adsorption

## Abstract

Preserving the structural integrity of biomimetic foldamers upon surface deposition is essential for their integration into functional molecular architectures and devices. When assembled in well-ordered monolayers, these molecules can exhibit distinctive characteristics. In this study, we investigate the electrospray-controlled ion beam deposition of foldamer molecules in an ultrahigh vacuum (UHV) environment on an Au(111) surface and examine how their conformation depends on the mean landing energy during deposition. At a low mean landing energy of about 0.6 eV, intact foldamers are observed on the surface, whereas higher landing energies predominantly result in unfolded molecules and partially folded states. Additionally, annealing of the substrate converts folded conformations into unfolded ones. These results highlight the importance of soft-landing conditions to maintain hydrogen-bond-stabilized architectures on surfaces, offering a model platform for studying the structure–function relationship of surface-supported thermolabile biomolecules.

## Graphical abstract

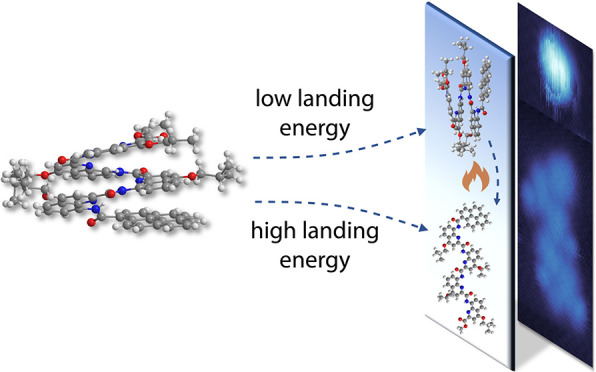

Numerous functionalities of macromolecules are driven by their two-dimensional (2D) and three-dimensional (3D) conformations. This concept is omnipresent in nature, as seen in proteins or DNA, and has inspired chemists to design macromolecules with well-defined 3D conformations to tailor conformation-related properties. Aromatic helical foldamers make up one of these synthetic molecular classes. Their potential applications range from molecular machinery^1,2^ and molecular recognition^3–6^ to charge transfer in molecular electronics.^7,8^ For example, helical oligoquinoline foldamers have been shown to promote unidirectional charge transport along their helices, thereby behaving as insulated molecular wires.^9^ In addition, the intrinsic chirality of helical molecules has been associated with polarized electron transport through chiral-induced spin selectivity,^10–12^ which is of interest for spin devices, quantum information processing, and novel quantum materials. Hence, achieving supported well-ordered 2D networks of these molecules may enable their future incorporation as monolayers in devices. There are reports of helical foldamer molecules on surfaces.^13,14^ However, their helical axes were often oriented parallel to the surface. The adsorption, self-assemblies, and properties of other helical molecules, for example helicenes, have been more extensively investigated on the surface.^15–18^ To develop a comprehensive understanding, a better control of the adsorption of foldamer molecules is clearly needed.

However, many of these macromolecules cannot be deposited intact and pure onto surfaces in ultrahigh vacuum (UHV) using conventional techniques such as organic molecular beam epitaxy. Other methods, such as drop casting, are often accompanied by a high level of contaminants. Electrospray ionization (ESI) is a technique used to transfer intact, charged molecules into the gas phase.^19^ Nondestructive ionization has been demonstrated for a variety of nonsublimable molecules, such as spin crossover complexes,^20,21^ nanoribbon precursors,^22–24^ molecules with thermally responsive side chains^25,26^ and biomolecules.^27–30^ Electrospray ion beam deposition (ESIBD) is an advanced technique that combines ESI with mass filtering and controlled landing-energy deposition onto surfaces under UHV conditions, enabling nondestructive adsorption with minimal contamination.^31–39^

A critical factor for the deposition of intact molecules under gentle ionization conditions is careful selection of the ion landing energy. In most cases, electrically conductive substrates are used, with an applied electricpotential controlling the ions’ landing energy. It is common practice to distinguish between the soft- and reactive-landing regimes. Soft landing refers to the retention of the molecular chemical structure,^36,40–42^ while reactive landing involves conformational changes, bond cleavage and chemical reactions.^43–45^

Anggara et al. investigated the deposition of coiled cellohexaose molecules on Cu(100) at landing energies ranging from 0.5 to 5 eV.^46^ They observed that at the lowest landing energy, the majority of molecules retained their gas-phase conformation, whereas at higher energies, open-chain molecules prevailed on the surface. Molecular dynamics simulations revealed a transfer of translational energy into other degrees of freedom (rotational, vibrational, and surface vibrational modes) resulting from collisions with the surface in the picosecond regime. Furthermore, the molecular conformation dynamics were found to depend on increases in the molecular vibrational energy. The different initial translational energies (0.5 and 5 eV) produced either insufficient or sufficient energy transfer to induce conformational changes, respectively. In another study, the influence of the substrate on the transfer of translational energy was examined.^47^ Freestanding graphene dispersed the translational energy of a protein within a few picoseconds, acting like a trampoline and enabling deposition without major conformational changes. Furthermore, for the deposition of Reichardt’s dye (RD) molecules on Cu(100) in the landing energy range of 2–50 eV,^43^ C–N bond cleavage was observed at landing energies of 5 eV and above. In this study, the influence of molecular orientation relative to the surface during collision was investigated by using molecular dynamics simulations of a positively charged RD molecule approaching a surface including image charge effects. Different molecular impact orientations were found to result in either molecular fragmentation or the deposition of intact RD species.

Previously, we have reported on the unfolding of the otherwise helical oligoamides of 8-amino-2-quinoline-carboxylic acid with a pyrene platform as foot (pyr-Q*_n_*, Figure 1b) upon deposition on Ag(111) with a landing energy of 4.5 eV, which can be hence ascribed to reactive landing.^48^ The term “unfolding” in the present context refers to an ordered flat ribbon-like surface conformation as opposed to “unfolding” of other macromolecules, such as DNA or certain proteins, implying disordered structures. This prompted the question of whether the metallic substrate or the collision impact caused the unfolding, since ion mobility (IM) measurements ruled out significant conformational changes induced by the electrospray process and confirmed intact helical foldamers in the gas phase.^48^

**Figure 1:**
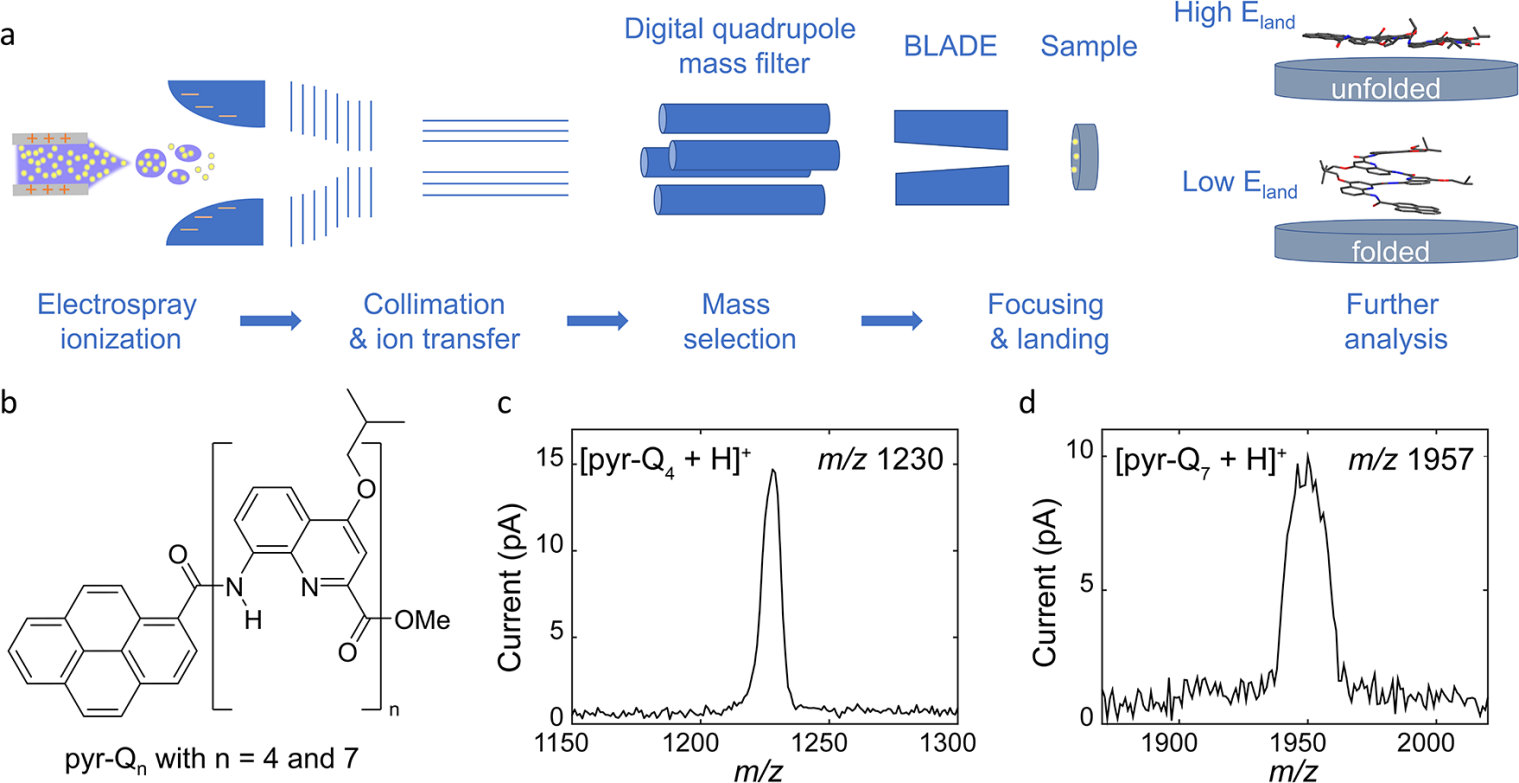
Electrospray ion beam deposition of foldamer molecules. (a) Scheme of the operational principle. (b) Molecular structure of pyr-Q*_n_*. Example of a mass spectrum of singly charged (c) [pyr-Q_4_ + H]^+^ (*m* = 1230 u) and (d) [pyr-Q_7_ + H]^+^ (*m* = 1957 u).

Here, we report on the room temperature (RT) deposition of both pyr-Q_4_ and pyr-Q_7_ with a custom-designed system designated ‘electrospray-controlled ion beam deposition (ES-CIBD)’^36^ on the Au(111) surface and on the parameters that determine the foldamers’ conformation upon adsorption (Figure 1a). The surface conformations of the foldamers were analyzed by low-temperature scanning tunneling microscopy (LT-STM) following deposition at RT and vacuum suitcase transfer. At very low landing energy, we were able to deposit significant proportions of pyr-Q_4_ and pyr-Q_7_ in a folded conformation onto the surface. Increasing the energy or annealing the decorated substrate entailed unfolding. Therefore, we identify the impact during deposition as a driving force for the unfolding of the helical molecules and we assess the landing energies and their influence on the molecular conformation. Due to their well-defined secondary structure stabilized by hydrogen-bonding, the foldamer molecules also offer a simple model system for studying fundamental aspects of biomolecular adsorption and structural integrity in UHV environments.

## Results and Discussion

To test the influence of the collision with the surface on the conformation, we first conducted deposition experiments with the smaller foldamer, pyr-Q_4_, sprayed in positive mode (see Methodology for details). Singly charged [pyr-Q_4_ + H]^+^ ions were mass selected by a digital quadrupole mass filter (*m*/*z* 1230, Figure 1c) and deposited on Au(111). The foldamer’s translational energy distribution in the ion beam was determined by measuring the deposition current as a function of the cutoff-energy (Figure 2a). The cutoff-energy, experimentally adjusted by the voltage drop between the last ion optics (BLADE) and the sample (Figure 1a), is defined as the energy where (nearly) no ion reaches the sample (see Methodology and SI for further information). Assuming a Gaussian translational energy distribution, we obtain a center energy at −2.4 eV and an energy width of 2.7 eV (FWHM) (Figure 2b).

To distinguish between the different depositions described in this study, we evaluate the mean landing energy. This value is defined as the mean translational energy of all ions reaching the surface. However, these values can be somewhat misleading due to the contribution of the energy distribution perpendicular to the direction of ion motion. To achieve precise energy control, narrowing the translational energy distribution is desirable. A more confined energy spread allows for a better definition of the landing energy, thereby improving reproducibility and minimizing unwanted unfolding upon impact. Several factors influence the narrowing of the energy distribution in the high-pressure stages of the ES-CIBD system. Other authors observed that the pressures in the early stages of ESIBD or introducing a buffer gas can play a significant role in energy thermalization.^49^ Nevertheless, a narrow kinetic energy distribution in the last vacuum region downstream, where ions still frequently scatter with residual gas, is crucial for the resulting energy distribution entering the quadrupole mass filter.^38^ Furthermore, optimization of the ion optics and mass filter parameters can further refine the energy distribution, enabling precise control over the ion landing conditions.

The first deposition of pyr-Q_4_ was performed with a mean landing energy of 1.9 ± 0.2 eV (Figure 2b). This is a 2.6 eV reduction in mean landing energy compared to the 4.5 eV in earlier experiments on Ag(111).^48^ Figure 2c shows an overview STM image of the Au(111) surface after deposition. The majority of the adsorbed pyr-Q_4_ molecules show similar features as previously on Ag(111), suggesting a similar unfolded surface conformation.^48^ We also reported on IM measurements, which implied that ESI itself does not unfold the molecules, and, hence, the impact on a metal substrate in UHV was causing the unfolding.^48^ Due to the low coverage, mostly unfolded single molecules with a few molecules forming dimers were observed (blue circle in Figure 2d). Interdigitation between the *iso*-butyl side chains of adjacentfoldamer molecules may promote dimer formation. This interaction motif is in good agreement with the observed self-assembly on Ag(111), similarly formed by dimers. In solution, the helical structure of the foldamers is stabilized through hydrogen bonding. Furthermore, the unfolding is hindered by repulsive interactions between the carbonyl oxygen atom and the quinoline endocyclic nitrogen atom at each quinoline-carboxamide linkage. These interactions render the helical structure of the foldamer molecules extremely stable in solution.^50,51^ On the other hand, hydrogen-bond energies are typically in the regime of several tenths of electron volts. Thus, most of the molecules in the ion beam should possess sufficient translational energy to break hydrogen bonds upon adsorption. However, whether the hydrogen bonds break or remain intact strongly depends on how efficient the energy is dissipated during surface collision, rather than solely on the amount of translational energy.^43,46,47^

**Figure 2:**
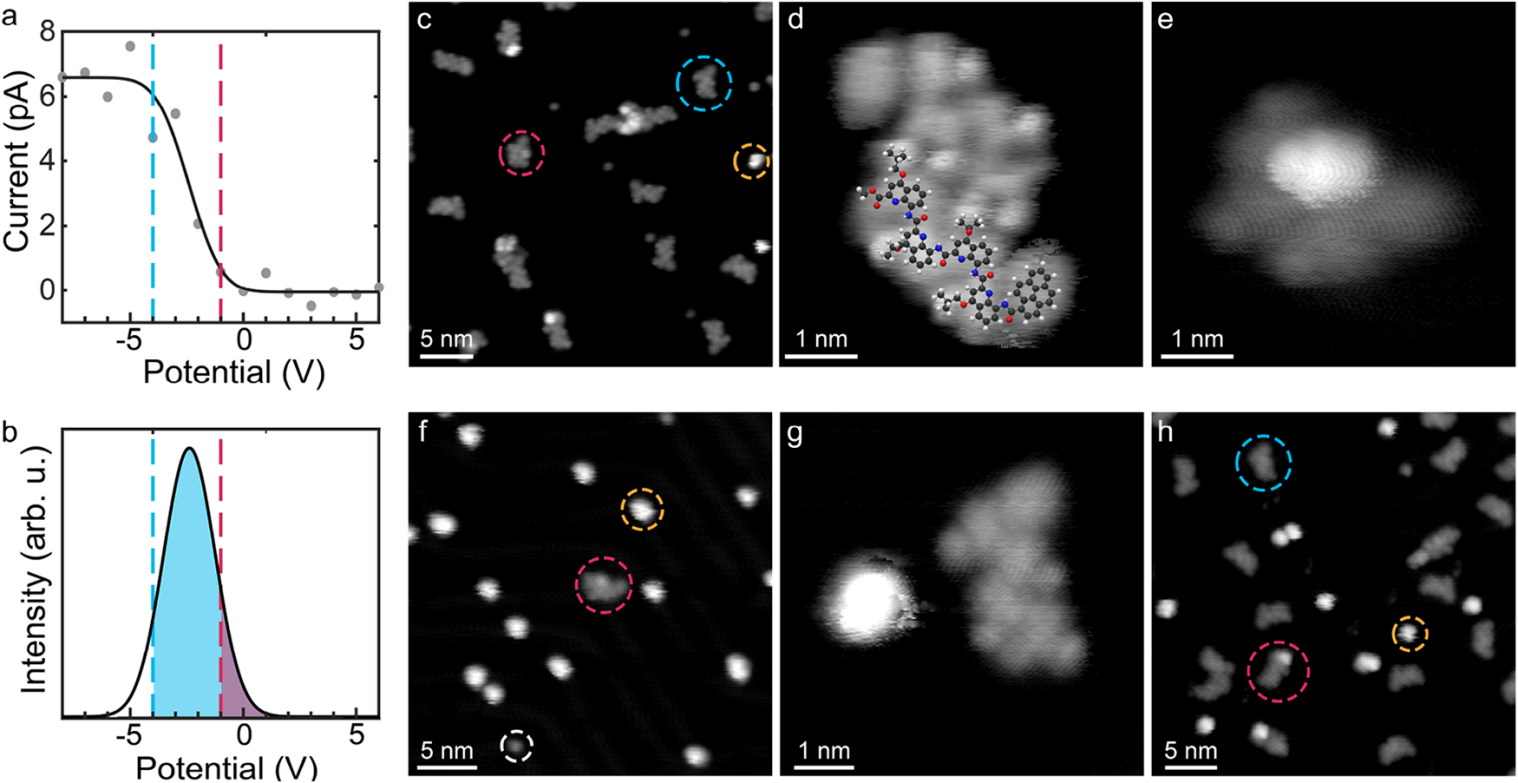
Pyr-Q_4_ depositions on Au(111). (a) Ion current measured on the sample as a function of the sample potential (gray dots). The black line represents the fit using a “Gaussian error function”. The sample potentials used for the “high” (blue dotted line) and “low” (magenta dotted line) landing energies are indicated. (b) Energy distribution of the ion beam (see Methodology and SI for more information). The cutoff-energy for the blue line corresponds to a “high” landing energy of 1.9 ± 0.2 eV, and the cutoff-energy for the magenta line corresponds to a “low” landing energy of 0.6 ± 0.2 eV. STM images of pyr-Q_4_ after high mean landing energy deposition of 1.9 ± 0.2 eV: (c) overview (−1000 mV, 20 pA), (d) a high-magnification of a pyr-Q_4_ dimer (−700 mV, 20 pA), and (e) an intermediate state between fully unfolded and folded (−300 mV, 40 pA). C, H, N, and O atoms are in gray, white, blue, and red. STM images after deposition with a low mean landing energy of 0.6 ± 0.2 eV: (f) overview (−400 mV, 20 pA) and (g) folded (left) and an unfolded (right) pyr-Q_4_ molecule coexisting (−50 mV, 100 pA). (h) Overview STM image of this surface after 10 min of annealing to 423 K (−500 mV, 30 pA). The molecules encircled in blue, magenta, and yellow correspond to unfolded, partially unfolded, and folded pyr-Q_4_ molecules, respectively. The white circle shows a contaminant.

In addition to unfolded molecules, a small amount of sligthly smaller surface species was observed (magenta circle in Figure 2c,e) with a length of about 2.5 nm. This species are approximately 1 nm smaller than a fully unfolded molecule (length: ∼3.5 nm, apparent height: ∼0.17 nm, Figure 2d) and feature a characteristic protrusion with an apparent height of about 0.3 nm, which is absent in the unfolded molecule.Considering the shape and increased apparent height, we assign these species to partially unfolded molecules. Besides these partly unfolded molecules a second species occurs even less frequently and appears as more compact objects with an apparent height of ∼0.34 nm and a diameter of approximately 2 nm (yellow circle in Figure 2c). This second surface speciesis associated with a fully folded molecule.

Intrigued by these observations, we decreased the mean landing energy to 0.6 ± 0.2 eV in a second preparation (Figure 2b) to investigate whether we could observe an increase in the occurrence of folded molecules. Indeed, STM measurements reveal a distinct change in the appearance of the surface species (Figure 2f and SI, Figure S2). Protrusions with a diameter of 2 nm and an apparent height of 0.34 nm were predominantly observed (yellow circle in Figure 2f), whereas only a few unfolded pyr-Q_4_ molecules existed (Figure 2f,g). Note that there are further protrusions with an even lower apparent height of just 0.15 nm (white circle in Figure 2f), identified as contaminants (see SI, Figure S3) from the vacuum suitcase transfer or other neutral adsorbents.

However, the STM observations of the folded pyr-Q_4_ surface species do not allow determination of whether the pyrene foot is adsorbed onto or lifted away from the surface, nor do they provide information about the handedness (right or left) of the helical conformation at the single-molecule level. In another study of an electrosprayed cyclic glucose oligomer, β-cyclodextrin, on Au(111), Grabarics et al. combined AFM imaging with theoretical calculations to successfully deduce distinct adsorption geometries.^52^ Remarkably, they were also able to resolve the orientation of hydroxyl groups and the presence of intramolecular hydrogen bonds.

The sample was subsequently annealed stepwise to 373, 423, 463, and 513 K for 10 min to investigate the thermal stability of the folded pyr-Q_4_. After the first annealing step to 373 K, we observed a significant increase in unfolded pyr-Q_4_ species (see SI, Figure S2) compared to the as-deposited sample, while the overall coverage was similar as before. After annealing to 423 K, unfolded pyr-Q_4_ surface species prevail (Figure 2h and SI, Figure S2). A further increase in temperature did not significantly affect the ratio between folded and unfolded molecules (see SI, Figure S2). After the thermal treatment at 513 K, the coverage of adsorbed molecules strongly decreased due to desorption (see SI, Figure S2). The ratios of unfolded, intermediate, and folded pyr-Q_4_ molecules for the different landing-energy depositions and after annealing to 423 K are summarized in Figure 3a. This evaluation shows that folded surface species observed after 0.6 ± 0.2 eV landing energy deposition can be thermally converted to the unfolded state of the foldamer molecule on the Au(111) surface. Overall, these findings substantiate the assignment of surface species identified by STM to the respective configurations.

**Figure 3:**
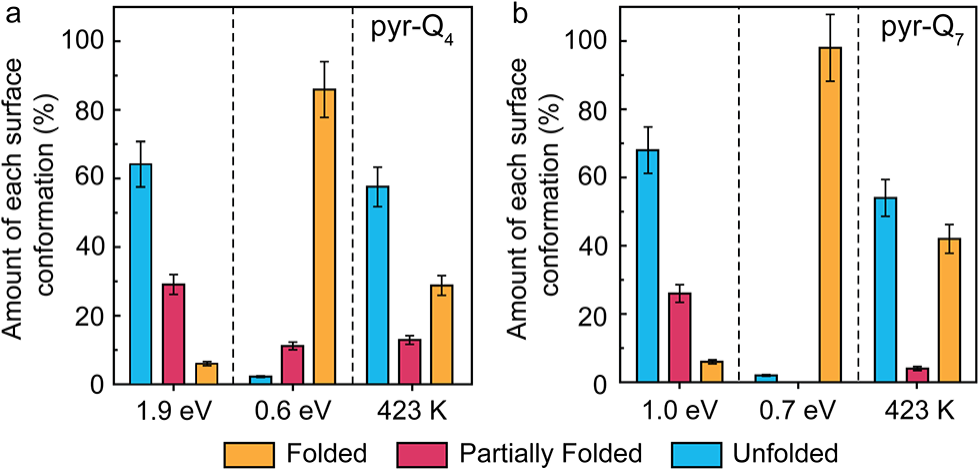
Statistical evaluation of surface conformations of (a) pyr-Q_4_ and (b) pyr-Q_7_ on Au(111). The folded, partially folded, and unfolded conformation of the foldamers on the terraces were counted for two landing energies and for the low landing energy after annealing to 423 K for 10 min (pyr-Q_4_) and for 30 min (pyr-Q_7_). For each preparation, at least 80 molecules were counted.

To gain further insights into the effect of adsorption impact on the conformation, we studied the longer foldamer pyr-Q_7_ (*m*/*z* 1957, Figure 1d). The preparation of the solution and the charging were carried out in the same way as for pyr-Q_4_. The current as a function of the sample potential and the corresponding fitted *dI/dV* curve are shown in Figure 4a,b, respectively. The Gaussian translational energy distribution has a center at −4.3 eV and an energy width of 4.4 eV (FWHM). After deposition with a mean landing energy of 1.0 ± 0.2 eV, pyr-Q_7_ is mostly present in the unfolded conformation (Figure 4c and the SI, Figure S4). Figure 4d shows a dimer in which the two molecules show an organization similar to that observed for the shorter foldamer. However, different unfolded surface conformations can be observed. This can be rationalized by the amount of different possible rotamers of pyr-Q_7_ obtained by rotation of the aryl-NH bonds and/or the aryl-carbonyl bonds.

**Figure 4:**
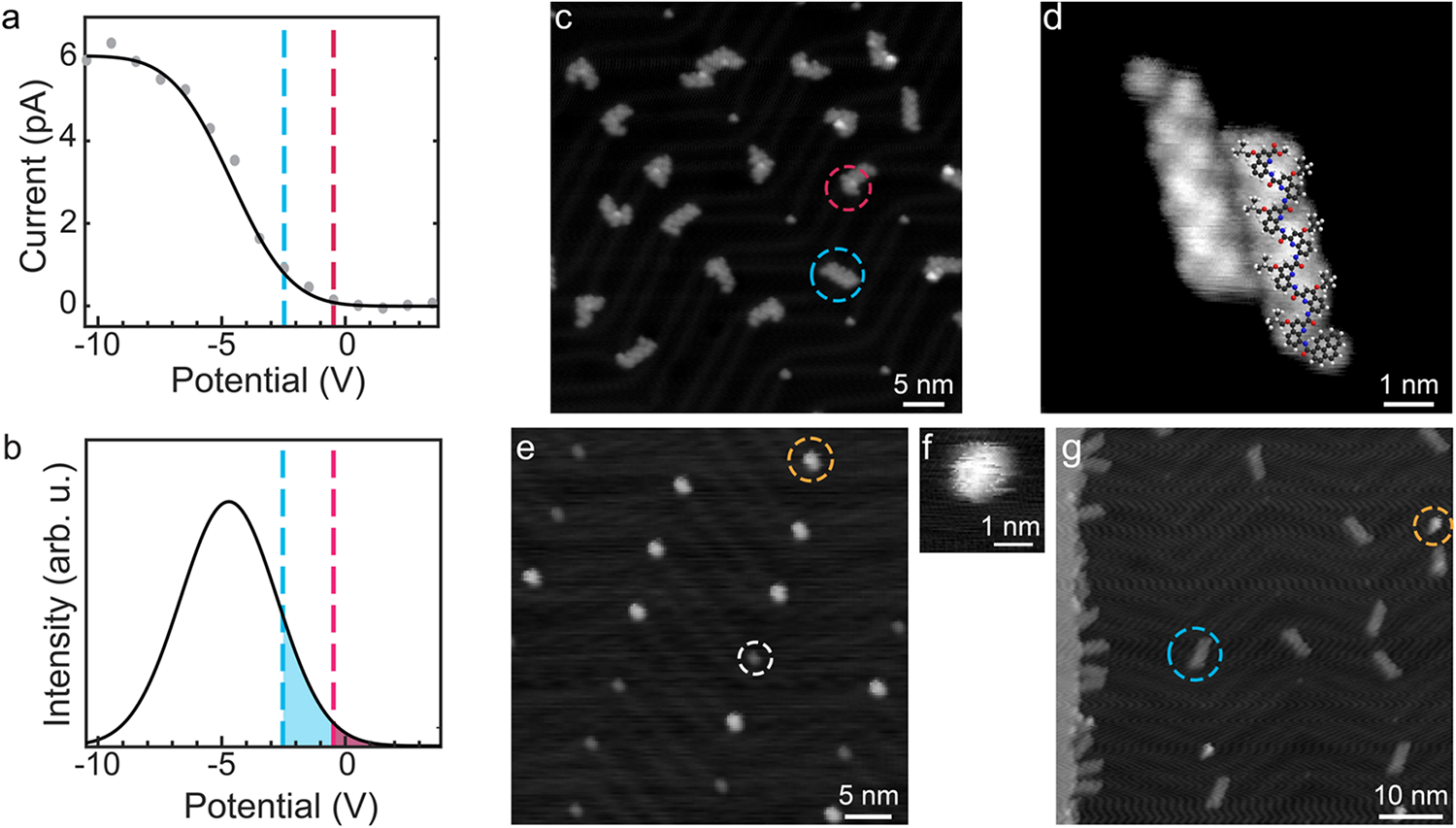
Pyr-Q_7_ depositions on Au(111). (a) Ion current measured on the sample as a function of the sample potential (gray dots). The black line represents the fit using a “Gaussian error function”. The sample potentials used for “high” (blue line) and “low” (magenta line) landing energies are indicated. (b) Energy distribution of the ion beam (see SI for more information). The cutoff-energy for the blue line corresponds to a “high” landing energy of 1.0 ± 0.2 eV, and the cutoff-energy for the magenta line corresponds to a “low” landing energy of 0.7 ± 0.2 eV. (c) Overview STM image of pyr-Q_7_ after deposition with a mean landing energy of 1.0 ± 0.2 eV (300 mV, 100 pA). (d) STM image of an unfolded pyr-Q_7_ dimer (−50 mV and 50 pA). C, H, N, and O atoms are in gray, white, blue, and red. (e) Overview STM image of pyr-Q_7_ with a lower mean landing energy of 0.7 ± 0.2 eV (1000 mV, 100 pA). (f) STM image of folded pyr-Q_7_ (−1000 mV, 100 pA). (g) Overview STM image after annealing to 423 K for 30 min (1000 mV, 100 pA). The molecules encircled in blue, magenta, and yellow correspond to unfolded, partially unfolded, and folded pyr-Q_7_ molecules, respectively. The white circle shows a contaminant.

After a deposition with a lower mean landing energy of 0.7 ± 0.2 eV (Figure 4b), we observed bright protrusions (yellow circle in Figure 4e,f) on the elbows of the Au(111) reconstruction and unfolded pyr-Q_7_ at step edges (see the SI, Figures S4 and S5). The sample was first annealed to 373 K to investigate thermal effects on the folded molecules. However, only folded molecules were observed on the terraces. We cannot entirely exclude the possibility that unfolded molecules diffused to the step edges, which are already decorated with unfolded and partially unfolded molecules. In a second step, the sample was annealed at 423 K for 30 min, where the largest increase in unfolded molecules appeared for pyr-Q_4_ experiments. The amount of bright protrusions decreased after this thermal treatment, and unfolded pyr-Q_7_ molecules were now also observed on the terraces (Figure 4g and SI, Figure S5). No aggregation of unfolded molecules was observed on the terraces. These observations suggest that intermolecular interactions do not contribute significantly to the thermal unfolding process. After annealing, the contaminants were no longer observed. Thus, we attribute the bright protrusions to the folded pyr-Q_7_ molecules, which unfold upon annealing, similar to the pyr-Q_4_ molecules. Notably, pyr-Q_7_ exhibits a higher thermal stability than pyr-Q_4_. Its thermally driven unfolding cannot be directly compared to the mechanochemical unfolding by surface collision since it certainly proceeds via different pathways.^53^

The statistical evaluation of the folded and unfolded conformation ratios of foldamer molecules on terraces is summarized in Figure 3b. The proportion of folded conformations undergoes a major change when comparing deposition at 1 ± 0.2 eV to that at 0.7 ± 0.2 eV. The folded species now becomes the predominant form on the terraces. However, it is worth noting that pyr-Q_7_ molecules remain mostly unfolded at step edges, even at low translational energies (see SI, Figures S4 and S5). Therefore, it cannot be ruled out that step edges act as active sites for the unfolding of pyr-Q_7_. If one assumes that the step edges are not active in the unfolding process and includes these molecules in the statistics, an approximate folded-to-unfolded/partially folded ratio of 6:4 is obtained. Molecules adsorbed at the step edges often cannot be clearly distinguished between partially unfolded and fully unfolded conformations; hence, they are grouped into the same category for this evaluation.

## Conclusions

We report on the influence of the mean landing energy on the surface conformation of two helical foldamer molecules with different lengths on Au(111) under UHV conditions. At a landing energy below 0.7 eV, the helical conformation of the foldamers was largely preserved following deposition at RT. Although it may seem surprising at first glance that both species (with a mass ratio of ca. 1.6) exhibit a similar dependence of the landing energy, this behavior may indicate that unfolding is triggered by a process during adsorption in which the energy is not efficiently distributed throughout the entire molecule. Annealing or choosing higher landing energies results in the unfolding of the helical structure of the foldamer. Remarkably, partially folded states were also observed. With this comparative study of landing energy profiles, we demonstrate a strong influence on the conformation of molecules. The next step should be a more detailed investigation of the landing geometry of the molecules, for example, using AFM as well as improved control of the orientation of even longer aromatic helices to achieve a well-ordered 2D assembly and investigate their properties, particularly charge transfer along the helix.

Further, these results shed light on possible approaches to conserve the intact secondary structure of a foldamer, opening up novel avenues to steer the behavior of adsorbed bio- or synthetic molecules with more complex 2D and 3D conformations. The ability to preserve the secondary structure of molecules will enable researchers to explore or exploit the conformation-dependent properties of these molecules in functional devices. Furthermore, the findings are useful for realizing the supported 2D assembly of molecules with controllable conformations.

## Methods

### ESIBD

The synthesis of the foldamer molecules was reported previously.^48^ The foldamers were dissolved in a mixture of acetonitrile (69 vol %), methanol (29 vol %), and acetic acid (2 vol %). The sprayed solutions had a concentration of 10^–4^ mol/L. The ESI emitter was a fused silica capillary with inner and outer diameters of 0.075 and 0.360 mm, respectively. The emitter voltage was set to ∼5 kV and the flow rate to 60–90 μL/h. Before each deposition, a mass spectrum was recorded to ensure pure deposition. The pressure during the depositions was below 5 × 10^–10^ mbar, and the sample was kept at room temperature during deposition. To ensure a high as possible reproducibility, several precautions were taken. First, identically prepared solutions were used for each deposition of pyr-Q_4_ and pyr-Q_7_, respectively. The ESI emitter voltage was set to 5 kV for all of the depositions. The temperature of the capillary at the vacuum interface was maintained at 350 K throughout the experiments. All parameters of the ion guides were kept constant within each series of pyr-Q_4_ and pyr-Q_7_ experiments, respectively.

### Sample Preparation

The Au(111) single-crystal surface was prepared *in situ* by multiple cycles of Ar^+^ sputtering and annealing to 650 K. The cleanliness of the substrate was assessed by STM. After depositing the molecule on Au(111), the sample was transferred with a vacuum suitcase (base pressure of 2 × 10^–10^ mbar) to an LT-STM.

### LT-STM

All STM measurements were performed by using a commercial Joule-Thomson STM (SPECS GmbH) with a chamber base pressure of 2 × 10^–10^ mbar. The tungsten tip was prepared through electrochemical etching. The tunneling bias is applied to the sample. The tunneling conditions for each STM image are given in the respective captions. All measurements were performed at 4.5 K. STM images were analyzed with the help of SpmImage Tycoon.^54^

## Supplementary Material



## Notes

An explanation of how the landing energy was determined, as well as additional STM data (PDF)

## ¶

School of Chemistry and Chemical Engineering, Nanchang University, Nanchang 330031, China
