# Supplementary material for: Landing-Energy-Controlled Surface Conformation of Electrosprayed Foldamer Molecules on Au(111)
Source: ACS Nano. 2026 Jan 18;20(4):3402–9. doi: 10.1021/acsnano.5c12973 (PMC12875027; doi:10.1021/acsnano.5c12973)
Supplement: Supplementary file 1 [file nn5c12973_si_001.pdf]

Supporting Information

Landing-Energy-Controlled Surface Conformation of Electrosprayed Foldamer Molecules on Au(111)

Shengming Zhang,[a] Dennis Meier,[a,b] Patrick Lawes,[c,d] Pengfei Zhao,[a] Jinhua Wang,[e] Victor Maurizot,[e] Andreas Walz,[a,f] Annette Huettig,[a,f] Hartmut Schlichting,[a,f] Anthoula C. Papageorgiou,[a,g] Joachim Reichert,[a]* Ivan Huc,[h,i]* and Johannes V. Barth[a,i]*

[a] Physics Department, E20, TUM School of Natural Sciences, Technical University of Munich, 85748 Garching, Germany

[b] Department of Chemistry, Tufts University, Medford, MA 02155, USA

[c] Institute of Nanotechnology and Institute of Quantum Materials and Technology (IQMT), Karlsruhe Institute of Technology, 76131 Karlsruhe, Germany

[d] Institut de Physique et de Chimie de Matériaux (IPCMS), Université de Strasbourg, UMR 7504, F-67034 Strasbourg, France

[e] CNRS, Bordeaux INP, CBMN, UMR5248, Univ. Bordeaux, F-33600 Pessac, France

[f] pureions GmbH, 82205 Gilching, Germany

[g] Laboratory of Physical Chemistry, Department of Chemistry, National and Kapodistrian University of Athens, Athens 157 71, Greece

[h] Department of Pharmacy, Ludwig-Maximilians-University Munich, 81377 Munich, Germany

[i] Cluster of Excellence e-conversion, 85748 Garching, Germany

Table of Contents

Determination of the landing energy 2
Figure S1: Geometry of ES-CIBD system during deposition 2
Figure S2: STM images of annealing steps of pyr-Q_4 6
Figure S3: STM images of control experiment 7
Figure S4: Overview STM images of pyr-Q_7 8
Figure S5: STM image of pyr-Q_7 decorated step edges 9
References 9

Determination of the landing energy

The translational energy distribution of the molecules in the ion beam was determined by measuring the ion current on the sample as a function of the sample potential. The principal geometry of the employed ES-CIBD system is described elsewhere.[1]

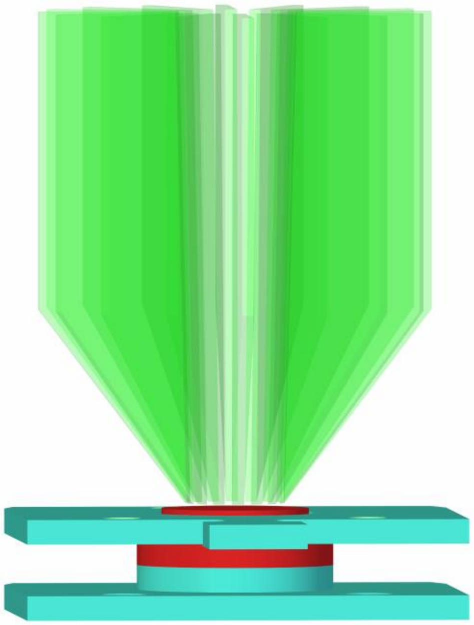

Figure S1. Geometry of the BLADE and sample during deposition.

Figure S1 shows the detailed situation close to the sample. The ions arrive from above through a special RF ion guide called BLADE (indicated in green). In short, it consists of 16 stainless

steel plates (0.5 mm thick) arranged in a circle around the flight axis of the ions, forming a slightly conical tube. The guide compresses the ion beam emerging from the QMF from a diameter of 7 to 4 mm. It is located directly in front of the sample. At its downstream end the distance between the plates is approximately 0.3 mm. The distance between the BLADE plates and sample is usually very small (0.2 mm by moving the sample). The sample is a stepped cylinder (indicated in red). The smaller diameter is typically 6 mm. It is fixed between two molybdenum plates (indicated in teal).

First, we investigate the influence of all RF-devices on the beam. In a homogeneous electric RF-field with the electric field strength E, the energy change ΔW of an ion with charge q and mass m is given by

$$\Delta W = \frac{q^2 E^2}{2m} \Delta t^2 + qE \sqrt{\frac{2W_0}{m}} \Delta t$$

within the time interval Δt. In the case of low initial energy W_0 the energy intake is proportional to $\Delta W \sim E^2 \Delta t^2$.

Table S1. Energy change ΔW of an ion for the different ion optics.

	Voltage	Frequency	Distance	$\sim\Delta W$	normed ΔW
QMF	1000 V	0.5 MHz	19 mm	$1108 \cdot 10^{-5}$	100%
SWIG	40 V	5 MHz	0.2 mm	$1.6 \cdot 10^{-5}$	0.14%
BLADE	60 V	4 MHz	0.3 mm	$2.5 \cdot 10^{-5}$	0.23%

Hence, the contribution of the QMF for the energy uptake is dominant. The last SWIG (in the high vacuum regime) contributes 0.14% to the energy change of an ion relative to the QMF, the BLADE contributes with 0.23%. Thus, the (Gaussian) energy distribution of an ion in the beam measured at the sample is exclusively caused by the QMF. Note that we have compared

the upper limits of energy which might be transferred to the ions. In reality this value is significantly lower for all RF-Guides due to statistical reasons and space charge effects.

Another factor contributing to a wider energy distribution might be the inhomogeneity of the electric field in translational direction. Any radial part of the mean field transfers energy from translation to radial direction. One possible cause of such inhomogeneities is the differences in the work functions of the materials used. The blade is made of stainless steel, the sample holder is made of molybdenum and the sample in the present case has a gold surface Au(111).

Nevertheless, in our case these potential differences in work function have no significant influence. The RF field typically keeps the ions away from the electrodes by at least the distance between the electrodes.[2] Thus, even when the ion beam expands after leaving the BLADE, the distance to the sample is short enough, so that the molybdenum has no influence.

Another contribution might come from the expanded ion beam being accelerated in the slit between BLADE electrodes and sample. Again, the small distance between sample and BLADE helps. Furthermore, the conducting sample surface short-circuits any electric field near to the sample. Thus, the ions are not significantly affected by the RF field, which decreases linearly toward the sample surface, as the beam expands after leaving the BLADE. Hence, the electric field between the BLADE outlet and the sample can be assumed to be homogeneous.

The cutoff-energy is defined as the sum of the translational energy of the ions leaving the BLADE and the voltage difference between the BLADE and the sample. The latter voltage accelerates the ions. More precisely it is the voltage difference between a) The Fermi-level corrected by the work function of the enclosing metal and b) the Fermi-level corrected by the work function of the sample. The (possibly different) work functions of both materials have no influence on the further argumentation as long as we assume homogeneous fields (no change in the radial direction) as shown before. In this case the work functions are eliminated by calibration.

The mean landing-energy is defined as mean translational energy perpendicular to the surface. As only ions with a velocity towards the sample may reach it, the energy-cutoff for integration is at zero energy. The energy distribution of the ions is assumed to be Gaussian. The zero point of energy is chosen to be at 1% intensity of the ion beam at the low side of the experimental Gaussian distribution. The landing-energy of the positive ion with $q = +1$ is calculated by integrating from

$$W = \frac{\int_{cut}^{+\infty} x f(x) dx}{\int_{cut}^{+\infty} f(x) dx}$$

where $f(x)$ is a Gaussian function defined as:

$$f(x) = e^{\frac{-(x-x_0)^2}{x_W}}$$

with the peak maximum of the Gaussian function x_0 and the FWHM energy $2\sqrt{ln2 \cdot x_W}$. Integration is started at the cutoff-energy cut.

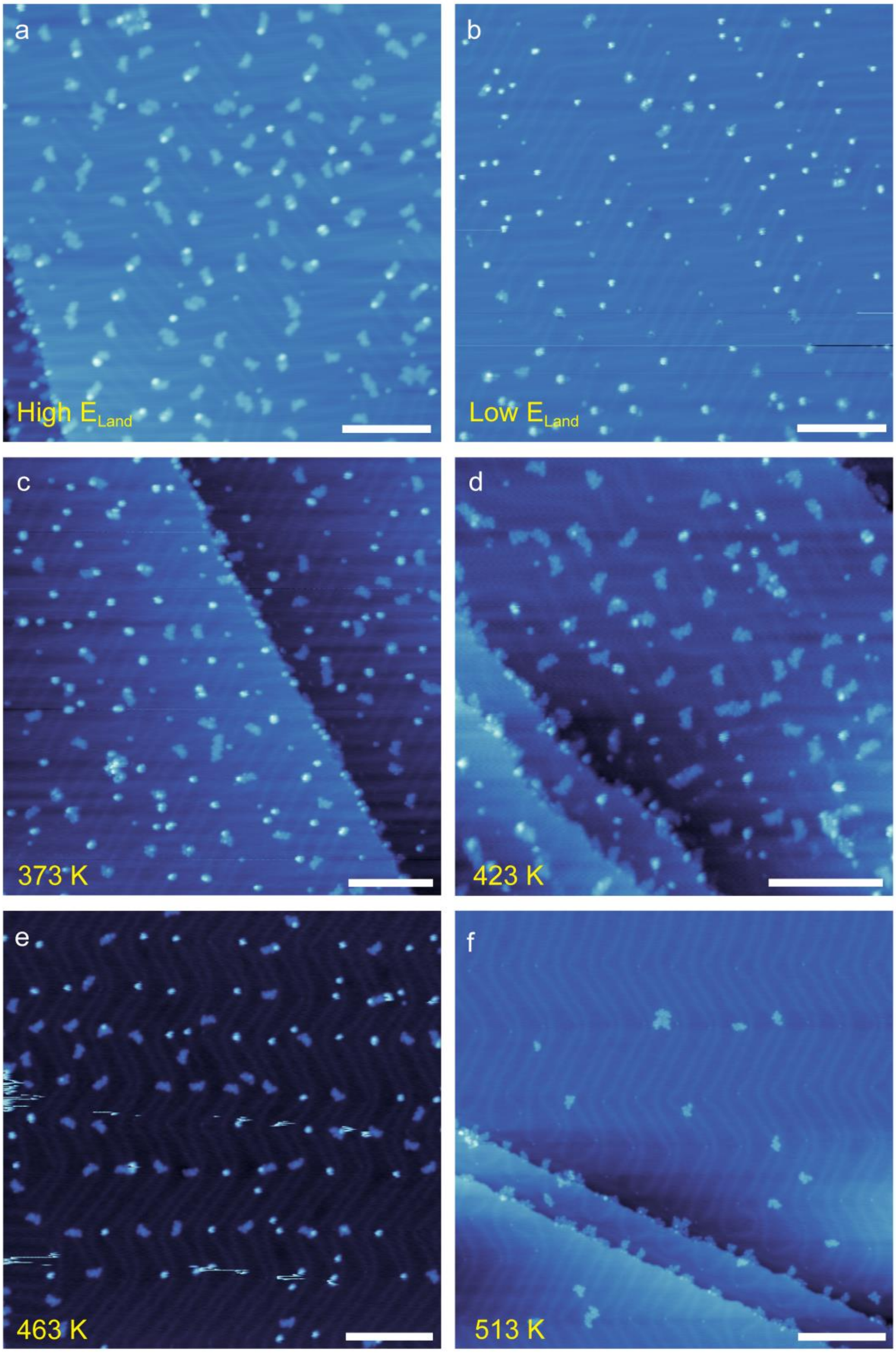

Figure S2. STM images of pyr-Q_4 deposited with (a) a landing energy of 1.9 eV (-1000 mV, 30 pA) and (b) a landing energy of 0.6 eV (-500 mV, 100 pA). STM images of the surface after the low landing energy deposition and an annealing to (c) 373 K (-1000 mV, 100 pA), (d) 423 K (-500 mV, 30 pA), (e) 463 K (-500 mV, 30 pA), and (f) 513 K (-500 mV, 40 pA) each for 10 minutes. Scale bars: 20 nm.

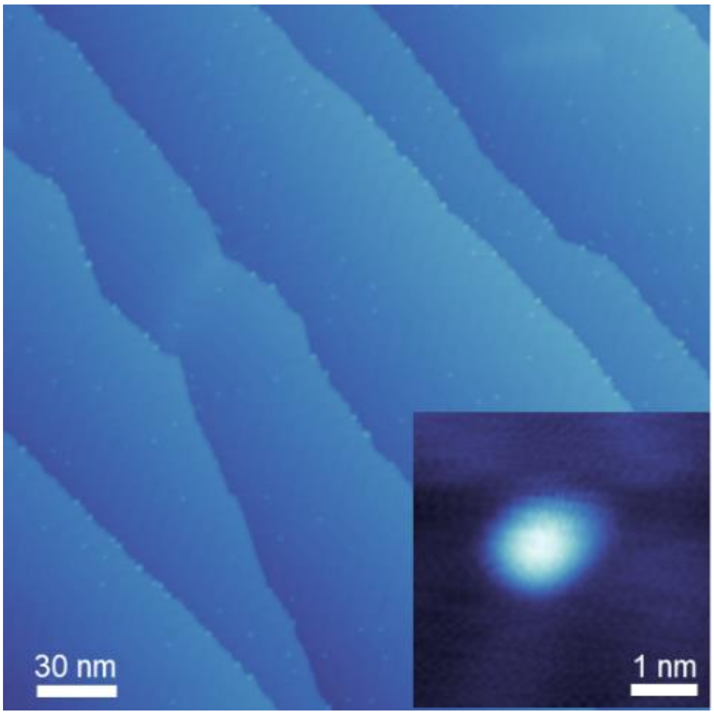

Figure S3. STM images of control experiment of a pyr-Q_7 deposition with a sample potential 20 V (both: -1000 mV, 100 pA). At this sample potential almost no charged pyr-Q_7 molecules reach the Au(111) surface. The features are therefore attributed to impurities either from neutrals in the deposition chamber or from the transfer via a vacuum suitcase from the ES-CIBD device to the LT-STM.

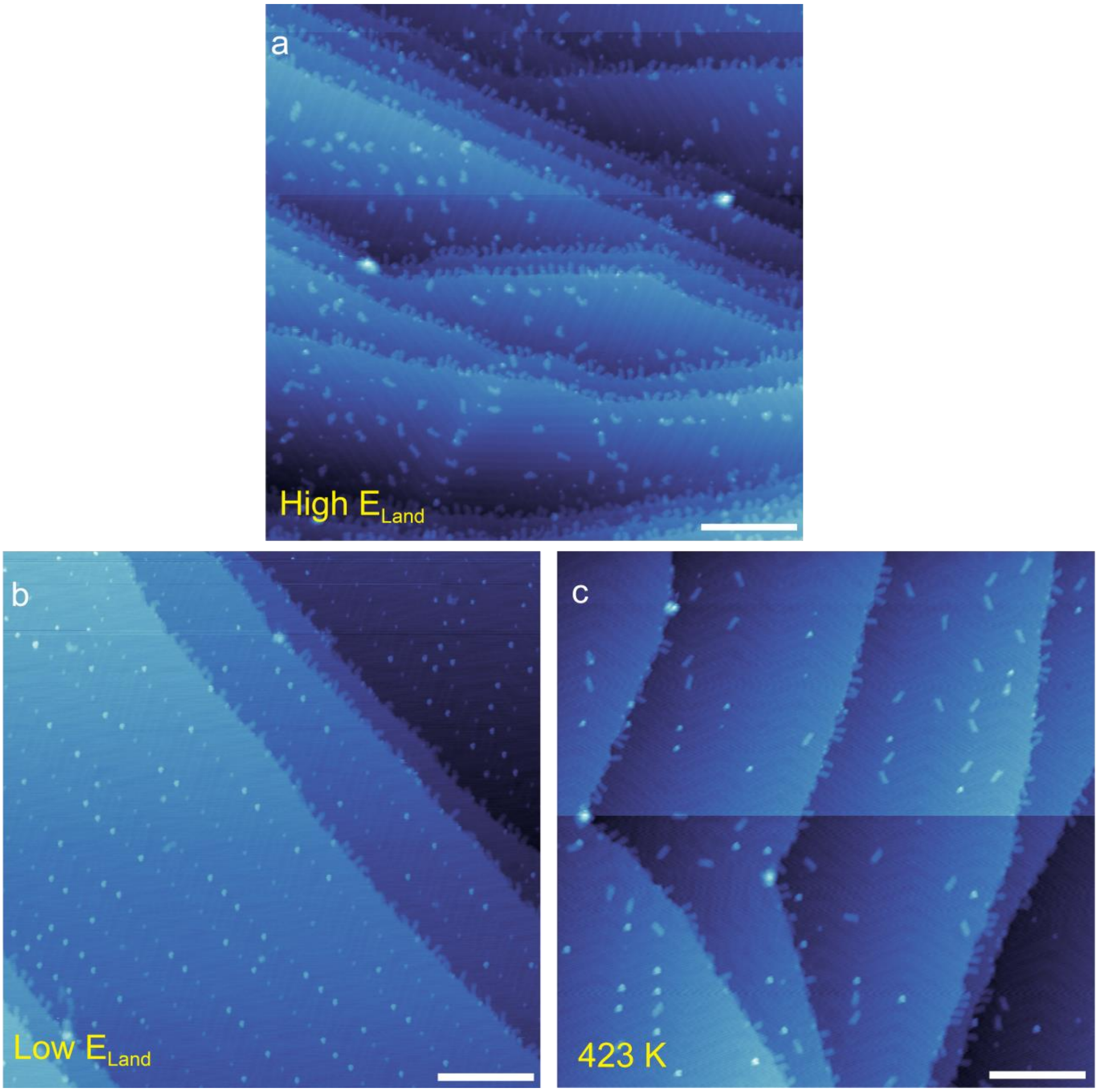

Figure S4. Large-scale STM images of pyr-Q_7 on Au(111) deposited with a mean landing energy of (a) 1 eV and (b) 0.6 eV and (c) after an annealing of the latter to 423 K for 30 min (all: -1000 mV, 100 pA). The folded molecules in (b) are observed after the annealing mostly in the unfolded conformation on the terraces in (c). Scale bars: 30 nm.

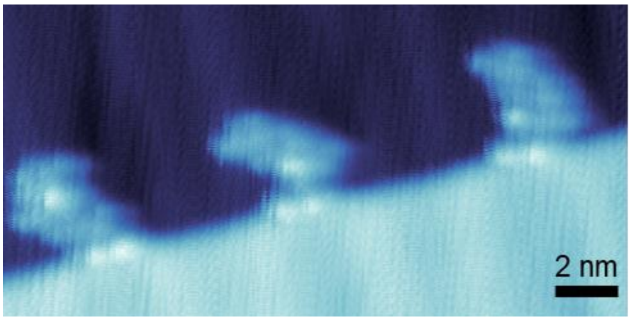

Figure S5. STM image of pyr-Q_7 at step edge (-1000 mV, 100 pA).

References

(1) Walz, A.; Stoiber, K.; Huettig, A.; Schlichting, H.; Barth, J. V. Navigate Flying Molecular Elephants Safely to the Ground: Mass-Selective Soft Landing up to the Mega-Dalton Range by Electrospray Controlled Ion-Beam Deposition. *Anal. Chem.* **2022**, *94* (22), 7767-7778.

(2) A. Walz, Doctoral thesis, Technische Universität München (München), 2020.
